# Decolorized Tincture of Iodine

**Published:** 1898-09

**Authors:** 


Decolorized Tincture of Iodine. A decolorized tincture of iodine does not contain free iodine, and it is, therefore, by no means as powerful a counter-irritant as the original tincture, which is a simple solution of iodine in alcohol. As a local discutient medical authorities state it to be of no more value than a solution of iodide of sodium or ammonium in water.—*Phar. Era.*

It is estimated that one-half of the world's production of quinine is consumed in the United States.

